# Patient Report and Review of Rapidly Growing Mycobacterial Infection after Cardiac Device Implantation 

**DOI:** 10.3201/eid2203.150584

**Published:** 2016-03

**Authors:** Varun K. Phadke, David S. Hirsh, Neela D. Goswami

**Affiliations:** Emory University, Atlanta, Georgia, USA

**Keywords:** Implantable cardioverter-defibrillator, cardiac pacemaker, nontuberculous mycobacteria, rapidly growing mycobacteria, *Mycobacterium fortuitum* group, bacteria, tuberculosis and other mycobacteria, bloodstream infections

## Abstract

Mycobacterial infections resulting from cardiac implantable electronic devices are rare, but as more devices are implanted, these organisms are increasingly emerging as causes of early-onset infections. We report a patient with an implantable cardioverter-defibrillator pocket and associated bloodstream infection caused by an organism of the *Mycobacterium fortuitum* group, and we review the literature regarding mycobacterial infections resulting from cardiac device implantations. Thirty-two such infections have been previously described; most (70%) were caused by rapidly growing species, of which *M. fortuitum* group species were predominant. When managing such infections, clinicians should consider the potential need for extended incubation of routine cultures or dedicated mycobacterial cultures for accurate diagnosis; combination antimicrobial drug therapy, even for isolates that appear to be macrolide susceptible, because of the potential for inducible resistance to this drug class; and the arrhythmogenicity of the antimicrobial drugs traditionally recommended for infections caused by these organisms.

## Abstract 2

As more of these devices are implanted, such infections are likely to be more frequently reported.

Infection is an uncommon but potentially devastating complication of cardiac implantable electronic device (CIED) implantation (*1*). Staphylococcal species cause most of these infections, followed by other pyogenic bacteria (*1*). CIED infections caused by mycobacteria have been reported infrequently; most of these infections result from the rapidly growing nontuberculous mycobacteria. We report a patient with an infection caused by a rapidly growing mycobacterium (RGM) in the *Mycobacterium fortuitum* group that developed following placement of an implantable cardioverter-defibrillator (ICD). We also review the published literature of cardiac device–associated infections caused by mycobacteria, focusing particularly on RGM.

## Patient Report

A 60-year-old man with chronic systolic heart failure, emphysema, and hepatitis C infection was admitted to Grady Memorial Hospital (Atlanta, Georgia, USA) with 2 days of purulent drainage from his ICD pocket site. Just before his admission, pain had developed, followed by spontaneous dehiscence (i.e., separation of the surgical incision along the suture line) of the pocket. He reported no fever or constitutional symptoms. Two months earlier, a single-chamber ICD had been inserted uneventfully in his left prepectoral region after he had an episode of ventricular fibrillation that led to cardiac arrest. At the time of device implantation, he had been receiving intravenous vancomycin and piperacillin/tazobactam for concomitant hospital-acquired pneumonia, and he was prescribed oral cephalexin for 5 days after the procedure. During outpatient follow-up visits at weeks 2 and 4 after device insertion, the patient’s wound was noted as unremarkable.

At the time of the new admission, the patient was afebrile with unremarkable vital signs. Physical examination showed mild, nontender edema over the ICD pocket; a 1-cm, shallow ulceration of the incision site; scant serous drainage; and minimal surrounding erythema. The patient had no peripheral stigmata of infective endocarditis, and the remainder of the examination was unremarkable. Laboratory studies showed a normal leukocyte count (5,500 cells/μL) with a slight (70%) neutrophil predominance. Blood cultures were collected, and he was started empirically on intravenous vancomycin for a suspected pocket infection. Transthoracic echocardiography was performed and showed no valvular vegetations. A transesophageal echocardiogram was deferred because of considerable laryngeal stenosis caused by traumatic endotracheal intubation at the time of his recent cardiac arrest.

On hospital day 2, the entire device, including the ICD generator and leads, was removed and sent for culture. Purulent fluid and necrotic tissue were noted in the pocket during explantation, and a drain was left in place at the time of wound closure (Figure, panel A). Gram stain of the pocket exudate showed beaded gram-positive rods. Within 5 days, the generator pocket tissue culture and 1 of 2 sets of blood cultures were growing an aerobic, gram-positive rod that also appeared beaded on Gram stain. Culture of the lead tip remained sterile. Because of the Gram-stain appearance of the blood culture isolate, an acid-fast stain was performed in the microbiology laboratory, and results showed the organism to be acid fast. At the request of the inpatient infectious diseases consultation service, the isolate was subcultured to mycobacterial growth media and was identified as an RGM. An HIV test result was negative. 

**Figure:**
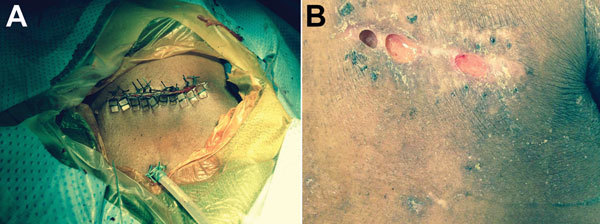
Photographs of the cardiac implantable electronic device pocket site for a 60-year-old man in whom infection developed at the implantation site of a cardiac implantable electronic device, Atlanta, Georgia, USA. A) Device pocket site after explantation. The wound was closed with pledged Ethibond sutures (Ethicon, Somerville, NJ, USA), and a Jackson-Pratt drain (closed-suction drainage system consisting of an internal drain connected by plastic tubing to a flexible bulb) was tunneled into the inferior aspect of the pocket. The drain was removed 24 hours postoperatively, and a small incision was left open to heal by secondary intention. B) Device pocket site 6 weeks after suture removal. Most of the incision healed well, with evidence of localized dehiscence (i.e., spontaneous partial separation of the surgical incision along the suture lines).

The patient’s antimicrobial drug regimen was empirically changed to intravenous cefoxitin, oral ciprofloxacin, and oral clarithromycin, a combination selected because of a clinical suspicion of *M. fortuitum* infection, surmised from the limited literature on RGM-associated CIED. Because of uncertainty about the capacity for outpatient therapeutic drug monitoring and because the patient had undergone surgical debridement and was thought to be clinically improved at the time of discharge, intravenous aminoglycoside therapy was deferred pending species identification and test results regarding antimicrobial drug susceptibility. A baseline electrocardiogram obtained before initiation of antimicrobial drugs showed a corrected QT interval (i.e., duration from start of Q wave to end of T wave in the heart’s electrical cycle) of 511 ms (reference <430 ms).

One week after device extraction, a computed tomography scan of the patient’s chest was performed, and results showed a residual, subclinical, peripherally enhancing, 2.7 × 1.0–cm collection of air and fluid over the left pectoralis muscle. Immediate device reimplantation was deferred. He was discharged on hospital day 14 with a wearable cardioverter-defibrillator to use until his physicians believed that device reimplantation was safe. The therapeutic plan at discharge was for at least 6 months of antimicrobial drug therapy, beginning with the cefoxitin, ciprofloxacin, and clarithromycin regimen and subsequently tailored to the organism and susceptibility profile when these data became available. 

Five weeks after device removal, the patient returned for outpatient follow-up and reported full adherence to his prescribed antimicrobial drug regimen. On examination, most of the wound over the previous device pocket had healed, but localized dehiscence and ongoing purulent drainage was evident (Figure, panel B). He was referred to plastic surgery for ongoing management of his wound. Identification and antimicrobial drug susceptibilities of the organism were still pending, so his antimicrobial drug regimen was not changed. Unfortunately, he was lost to follow-up; 2 months later, he discontinued use of his wearable cardioverter-defibrillator and died of out-of-hospital cardiac arrest.

By use of high-performance liquid chromatography, the patient’s isolate was identified as a *Mycobacterium fortuitum* group organism. Further genetic testing for species-level identification was not performed. The following susceptibilities were identified: cefoxitin intermediate (MIC 64 μg/mL), ciprofloxacin susceptible (MIC <0.12 μg/mL), and clarithromycin resistant (MIC 8 μg/mL after 14 days of incubation) (Table 1).

**Table 1:** Antimicrobial drug susceptibility profile of patient’s *Mycobacterium fortuitum* group isolate*

Antimicrobial drug	MIC,** μ**g/mL	Interpretation*
Amikacin	<1	Susceptible
Cefoxitin	64	Intermediate
Ciprofloxacin	<0.12	Susceptible
Clarithromycin	8	Resistant†
Doxycycline	>16	Resistant
Imipenem	8	Intermediate
Linezolid	2	Susceptible
Moxifloxacin	<0.25	Susceptible
**T**igecycline	0.12	‡
Trimethoprim/sulfamethoxazole	1/19	Susceptible
Tobramycin	>16	Resistant

*According to breakpoints defined by the Clinical and Laboratory Standards Institute (*2*). †Clarithromycin MIC after 14 d of incubation. ‡No accepted breakpoints from the Clinical and Laboratory Standards Institute exist for tigecycline.

## Discussion

The RGM species are ubiquitous environmental organisms that have been isolated from soil, food, natural and municipal water, various plants and animals, and hospital surfaces (*3*). These organisms are not believed to be permanent members of the human bacterial flora but often become transient colonizers after frequent exposure. The most commonly encountered RGM species in clinical practice are *M. abscessus*, *M. chelonae*, and *M. fortuitum*, but >100 species have been identified (*3*). These organisms are capable of growth on standard mycobacterial (e.g., Middlebrook 7H11 or Lownstein-Jensen) and routine bacteriologic (e.g., sheep’s blood and MacConkey agar) growth media. However, colonies may take >5 days to appear on standard media, exceeding the incubation time of routine cultures in many clinical microbiology laboratories. Even when growth is observed, these organisms often appear as beaded gram-positive bacilli on routine Gram stain and may be misidentified as contaminants (*4*). Therefore, a high index of suspicion for a potential RGM infection is needed for an accurate diagnosis.

Despite increasing recognition that these organisms can cause infections associated with prosthetic devices and surgical sites, RGM infections complicating implanted cardiac devices are still uncommon. We searched the available literature using PubMed with no starting date restrictions through March 31, 2015, and identified only 32 previously reported cases of CIED infections caused by any mycobacterial species. Including our patient, 23 (70%) of 33 reported infections were caused by an RGM species (*5**–**24*) (Table 2). We found 2 reports of CIED infections caused by *M. avium* complex (*26**,**27*) and 8 reports of infections caused by *M. tuberculosis* complex organisms (*28**–**33*). Of the 23 RGM infections, 21 (91%) were reported in the past 10 years, a trend likely resulting from improvements in microbiologic techniques and increased recognition of these organisms as causative pathogens. Mean age of case-patients with RGM infections was 65.4 years, consistent with age trends for CIED implantation. Sixteen (70%) case-patients had infections associated with permanent pacemakers. Among 21 case-patients for which time of onset was reported, 5 (24%) infections developed >6 months after the most recent device manipulation. Although cardiac devices can become secondarily infected because of seeding from incidental bloodstream infections, the RGM species are uncommon causes of bacteremia. Instead, the source of early-onset CIED infections is more likely inoculation of the organism into the pocket at the time of the implantation procedure. This source contrasts with the probable source for the 8 reported CIED infections caused by *M. tuberculosis* complex. Manifesting >11 months after device manipulation, these infections more likely resulted from reactivation disease, mycobacteremia, and secondary seeding of the device.

**Table 2:** Clinical and demographic information for published cases of cardiac device infections due to rapidly growing mycobacteria*

Year (ref)	Age, y/ sex	Organism	Type	Onset†	Bacteremia/ lead infection‡	IE§	Macrolide resistant	Device removed	Antimicrobial drug therapy	Outcome
*Mycobacterium fortuitum* group										
1998 (*6*)	74/M	*M. fortuitum* + *M. chelonae*	PPM	13 d	NR/NR	NR	NR	Yes	FQ + AG × 4 wk	Cured
2005 (*9*)	62/F	*M. fortuitum*	PPM	6 mo	Yes/yes	Yes	No	Yes	CLR + CIP × 4 wk, DOX + CIP × 24 wk	Cured
2005 (*10*)	74/M	*M. peregrinum*	ICD	6 wk	Yes/NR	NR	No	Yes	CLR + CIP × 6 wk	Cured
2005 (*8*)	72/M	*M. fortuitum*	PPM	2 wk	No/NR	No	Yes	Yes	CIP + AG × 2 wk, CIP × 6 mo	Cured
	61/M	*M. fortuitum*	ICD	17 mo	No/yes	No	Yes	Yes	LVX × >1 y¶	Cured
2006 (*11*)	80/M	*M. fortuitum*	PPM	18 d	Yes/NA	No	No	No	CLR + CIP × 6 wk	Cured
2007 (*13*)	84/F	*M. fortuitum*	PPM	1 mo	No/no	No	Yes	Yes	LVX × 3 mo	Cured
2007 (*15*)	78/F	*M. fortuitum*	PPM	3 mo	Yes/yes	No	NR	Yes	CLR + LVX + LZD × 2 wk, CLR + LVX × 6 mo	Cured
2007 (*16*)	78/F	*M. fortuitum*	PPM	<4 mo	Yes/NR	NR	NR	Yes	CLR + LVX + LZD × 2 wk, CLR + LVX × 22 wk	Cured
	77/F	*M. mageritense*	PPM	3 wk	NR/NA	NR	NR	No	FQ × 6 mo	Cured
2009 (*18*)	15/F	*M. fortuitum*	PPM	7 wk	Yes/yes	No	No	Yes	CLR + CIP × 6 mo	Cured
2010 (*20*)	78/M	*M. fortuitum*	PPM	NR	Yes/NR	NR	NR	Yes	CLR + CIP × 26 wk	Cured
2012 (*23*)	43/M	*M. fortuitum*	ICD	4 y	Yes/yes	Yes	No	Yes	CLR + CIP + AG	Died
2012 (*22*)	75/M	*M. peregrinum*	PPM	1 y	Yes/yes	No	NR	Yes	CLR + CIP × mo	Cured
2015#	60/M	*M. fortuitum* group	ICD	6 wk	Yes/no	No	Yes	Yes	CLR + CIP + FOX	Died
*M. abscessus* complex										
1998 (*5*)	68/M	*M. abscessus*	PPM	19 y	NR/yes	NR	No	Yes	CLR + AG + FOX × 5 wk	Died
2005 (*7*)	53/M	*M. abscessus*	ICD	2 wk	NR/NR	NR	Yes	Yes	CLR × 24 wk	Cured
2007 (*14*)	43/F	*M. massiliense*	PPM	11 mo	NR/yes	NR	No	Yes	CLR × 6 mo	Cured
*M. smegmatis* complex										
2006 (*12*)	86/M	*M. goodii*	PPM	16 d	Yes/NR	NR	NR	Yes	Multiple, ending with MIN + AG × 2 wk	Cured
2008 (*17*)	85/M	*M. goodii*	ICD	<7 d	No/NR	No	NR	Yes	TMP/SXT × 8 wk	Cured
2009 (*19*)	23/M	*M. goodii*	PPM	8 d	No/NA	No	Yes	No	DOX + FQ × 6 mo	Cured
*M. chelonae* complex										
2014 (*24*)	63/M	*M. chelonae*	PPM	NR	No/yes	Yes	NR	Yes	CLR + LVX + AG × >2 mo	Cured
Ungrouped rapidly growing species										
2011 (*21*)	73/M	*M. phlei*	ICD	1 mo	No/NR	NR	No	Yes	SXT + DOX × 12 mo	Cured

*AG, aminoglycoside; CIP, ciprofloxacin; CLR, clarithromycin; DOX, doxycycline; FOX, cefoxitin; FQ, fluoroquinolone other than CIP or LVX; ICD, implantable cardioverter-defibrillator; IE, infective endocarditis; LVX, levofloxacin; LZD, linezolid; MIN, minocycline; NA, not available; NR, not reported; PPM, permanent pacemaker; ref, reference; TMP/SXT, trimethoprim/sulfamethoxazole. †Time since most recent device manipulation. ‡Defined as positive lead culture, acid fast stain, or presumptive diagnosis on the basis of imaging or operative findings. §Transthoracic or transesophageal echocardiographic findings as defined by the Duke criteria (*25*). ¶No end date for therapy was specified, but patient had at least 1 year of treatment. #The patient described in this article.

The most commonly isolated organisms have been in the *M. fortuitum* group, which account for ≈50% of mycobacterial CIED infections in patients and nearly two thirds of infections caused by an RGM species. The *M. fortuitum* group has historically included *M. fortuitum* and *M. peregrinum*, although *M. mageritense* and others have also been proposed as members of this group (*3*). The preponderance of *M. fortuitum* infections among patients with cardiac device implantations mirrors trends observed for poststernotomy (*34*) and postaugmentation mammoplasty (*35*) infections caused by RGM. Although most skin and soft tissue infections caused by RGM, particularly after surgical or nonsurgical trauma, result from *M. fortuitum* (*3*), this organism is not considered a normal skin commensal. Sources of these infections are instead thought to be largely environmental (*3*). Nevertheless, among all skin and soft tissue infections caused by RGM, those on the chest or back seem more likely to result from *M. fortuitum* than from other RGM species (*36*).

Another trend we observed was that 11 (48%) of the 23 patients with a RGM infection had associated mycobacteremia (5 had no reported blood culture results). This finding indicates that the infection had spread beyond the device pocket to the intravascular component of the CIED system, suggesting endovascular infection. In the 13 (57%) patients for which both blood culture results and echocardiographic findings were reported, 4 (31%) had device-related endocarditis, as defined by the Duke criteria (*9**,**18**,**23**–**25*). Three of these 4 patients fulfilled clinical criteria for infective endocarditis on the basis of echocardiographic findings; the fourth had no echocardiographic abnormalities but fulfilled pathologic criteria on the basis of isolation of the organism in an operative culture. This patient was the only one with valvular endocarditis among all the reports in our review (*18*). 

Conversely, in 4 patients, including the patient described in this article, mycobacteremia was detected in the absence of echocardiographic abnormalities. The patient we describe had an unremarkable transthoracic echocardiogram, but a transesophageal echocardiogram could not be performed for definitive evaluation of CIED-related endocarditis. Overall, the low rates of valvular endocarditis or disseminated infection suggest that CIED infections caused by an RGM behave similarly to catheter-related bloodstream infections caused by these organisms (*37*), although severe complications of CIED infections associated with bacteremia have rarely been described (*18**,**23*).

The Clinical and Laboratory Standards Institute recommends routine broth microdilution susceptibility testing of all RGM isolates against amikacin, cefoxitin, ciprofloxacin, clarithromycin, doxycycline, imipenem, and sulfamethoxazole (or trimethoprim/sulfamethoxazole), but newer agents (e.g., linezolid, moxifloxacin, and tigecycline) also frequently show in vitro activity against these organisms (*2*). Of all RGMs, the *M. fortuitum* group is traditionally considered the most susceptible to antimicrobial drugs, with isolates frequently being susceptible to many agents tested. However, current guidelines by the American Thoracic Society and the Infectious Diseases Society of America recommend therapy with >2 active drugs for several months for optimal results. The isolate of the patient in this report showed resistance to several tested agents, including clarithromycin (Table 1). Among patients with CIED infections caused by *M. fortuitum* group organisms for which susceptibility data were reported, only 4 (including the patient reported in this article) had macrolide resistance. A previous review of the RGM similarly noted that most *M. fortuitum* clinical isolates were macrolide susceptible by in vitro methods (*3*).

Genetic studies published after that review revealed that most, if not all, *M. fortuitum* isolates also harbor an *M. fortuitum* rRNA methylase gene, termed *erm*(39), that, if active, can confer macrolide resistance (*38**,**39*). Although the clinical significance of this potential mechanism of inducible macrolide resistance is unclear, particularly in strains in which the gene is inactive at baseline, this finding has led many experts to advise caution to clinicians who consider prescribing macrolide-based regimens for serious *M. fortuitum* infections, even when the isolate is reported as susceptible to macrolides on the basis of broth microdilution methods (*2*). Nevertheless, in most reported CIED infections caused by macrolide-susceptible *M. fortuitum* group organisms, a macrolide and fluoroquinolone combination has been used successfully. We speculate that the patient described in this article experienced delayed wound healing resulting from inadequate activity of the empirical antimicrobial regimen against his isolate.

Given the recommended antimicrobial drug regimens for infections caused by an RGM, cardiac device infections resulting from these organisms can pose a unique therapeutic dilemma. On the one hand, the propensity to biofilm formation makes these organisms difficult to eradicate with antimicrobial drug therapy alone. Consequently, most experts advocate an extended course of antimicrobial drugs combined with device removal (*37*), the strategy used in all but 3 (13%) of the 23 previously described case-patients with infections caused by an RGM. On the other hand, some of the most active agents against this group of organisms belong to the macrolide or fluoroquinolone classes, which are both types of antimicrobial drugs with potential proarrhythmic effects (*39**,**40*). An antimicrobial drug combination that has the potential to precipitate arrhythmias becomes problematic in patients being considered for device removal and having a preexisting risk for conduction abnormalities (caused by an underlying conduction disease, cardiomyopathy, or concomitant proarrhythmic medications). The patient we describe was noted to have a prolonged corrected QT interval before the start of macrolide or fluoroquinolone therapy, a circumstance that made selection of an appropriate empirical antimicrobial regimen challenging. This patient highlights the importance of expedited antimicrobial drug susceptibility testing for managing these infections, including evaluation of newer antimicrobial drugs with fewer direct arrhythmogenic effects than those resulting from macrolides or fluoroquinolones.

Infections occurring after implantation of cardiac devices and caused by nontuberculous mycobacteria are uncommon, but as more devices are implanted, such infections are likely to be more frequently reported. Our patient illustrates many of the common clinical features of postimplantation CIED infections caused by RGMs, including early onset (<6 months from the most recent manipulation of the device) of disease, initial identification of the organism as a gram-positive bacillus, and isolation of a *M. fortuitum* group organism as the causative pathogen. In addition, this patient highlights several unique issues that warrant further investigation, such as reliability of macrolide therapy for *M. fortuitum* group infections and safety of long-term macrolide and fluoroquinolone use in patients with a preexisting high risk for serious arrhythmias.
